# Coffee Consumption and Non-alcoholic Fatty Liver Disease: An Umbrella Review and a Systematic Review and Meta-analysis

**DOI:** 10.3389/fphar.2021.786596

**Published:** 2021-12-13

**Authors:** Chayanis Kositamongkol, Sukrit Kanchanasurakit, Chiraphong Auttamalang, Nutkamon Inchai, Thanatchaporn Kabkaew, Sarunporn Kitpark, Nathorn Chaiyakunapruk, Acharaporn Duangjai, Surasak Saokaew, Pochamana Phisalprapa

**Affiliations:** ^1^ Division of Ambulatory Medicine, Department of Medicine, Faculty of Medicine Siriraj Hospital, Mahidol University, Bangkok, Thailand; ^2^ Division of Pharmaceutical Care, Department of Pharmacy, Phrae Hospital, Phrae, Thailand; ^3^ Division of Clinical Pharmacy, Department of Pharmaceutical Care, School of Pharmaceutical Sciences, University of Phayao, Phayao, Thailand; ^4^ Center of Health Outcomes Research and Therapeutic Safety (Cohorts), School of Pharmaceutical Sciences, University of Phayao, Phayao, Thailand; ^5^ Unit of Excellence on Clinical Outcomes Research and IntegratioN (UNICORN), School of Pharmaceutical Sciences, University of Phayao, Phayao, Thailand; ^6^ Unit of Excellence on Herbal Medicine, School of Pharmaceutical Sciences, University of Phayao, Phayao, Thailand; ^7^ Department of Pharmacotherapy, College of Pharmacy, University of Utah, Salt Lake City, UT, United States; ^8^ Department of Physiology, School of Medical Sciences, University of Phayao, Phayao, Thailand; ^9^ Novel Bacteria and Drug Discovery Research Group, Microbiome and Bioresource Research Strength, Jeffrey Cheah School of Medicine and Health Sciences, Monash University Malaysia, Bandar Sunway, Malaysia; ^10^ Biofunctional Molecule Exploratory Research Group, Biomedicine Research Advancement Centre, School of Pharmacy, Monash University Malaysia, Bandar Sunway, Malaysia

**Keywords:** coffee, non-alcoholic fatty liver disease, liver fibrosis, umbrella review, meta-analysis

## Abstract

**Background:** The effects of coffee consumption on hepatic outcomes are controversial. This study investigated the associations between coffee consumption and the incidence of non-alcoholic fatty liver disease (NAFLD) in the general population and the reduction of liver fibrosis among patients with NAFLD.

**Methods:** The study consisted of two parts: an umbrella review and a systematic review and meta-analysis (SRMA). The searches for each part were performed separately using PubMed, EMBASE, Cochrane, Scopus, and CINAHL databases. All articles published up to September 2021 were reviewed. To be eligible, studies for the umbrella review were required to report outcomes that compared the risks of NAFLD in the general population and/or liver fibrosis in patients with NAFLD who did and did not drink coffee. Our SRMA included primary studies reporting the effects of coffee consumption on NAFLD-related outcomes. The outcomes were pooled using a random-effects model and reported in both qualitative and quantitative terms (pooled risk ratio, odds ratio, and weighted mean difference).

**Results:** We identified four published SRMAs during the umbrella review. Most studies showed that individuals in the general population who regularly drank coffee were significantly associated with a lower NAFLD incidence than those who did not. Our SRMA included nine studies on the effects of coffee consumption on NAFLD incidence. Pooled data from 147,875 subjects showed that coffee consumption was not associated with a lower NAFLD incidence in the general population. The between-study heterogeneity was high (*I* ^2^, 72–85%). Interestingly, among patients with NAFLD (5 studies; *n* = 3,752), coffee consumption was significantly associated with a reduction in liver fibrosis (odds ratio, 0.67; 95% CI, 0.55 to 0.80; *I* ^2^, 3%). There were no differences in the coffee consumption of the general population and of those with NAFLD (4 studies; *n* = 19,482) or by patients with no/mild liver fibrosis and those with significant fibrosis (4 studies; *n* = 3,331).

**Conclusions:** There are contrasting results on the effects of coffee on NAFLD prevention in the general population. Benefits of coffee consumption on liver fibrosis were seen among patients with NAFLD.

**Systematic Review Registration**: https://www.crd.york.ac.uk/prospero/display_record.php?ID=CRD42021226607, identifier CRD42021226607

## Introduction

Non-alcoholic fatty liver disease (NAFLD) is a global chronic health problem. Meta-analyses have reported a prevalence ranging between 25 and 32% (Younossi et al., 2016; Park et al., 2021). The incidence of NAFLD has tended to increase over time due to behavioral changes leading to unhealthy lifestyles in the global population (European Association for the Study of the Liver, European Association for the Study of Diabetes, European Association for the Study of Obesity, 2016; Li et al., 2019; Calabrò et al., 2020). The latest meta-analyses showed that the incidence of NAFLD was approximately 42.8–50.9 per 1,000 person-years (Li et al., 2019; Park et al., 2021). Based on current evidence, NAFLD can lead to many serious complications. They include hepatic-related events (such as liver cirrhosis, end-stage liver disease, and hepatocellular carcinoma) and non-hepatic-related events (for example, cardiovascular and chronic kidney diseases) (European Association for the Study of the Liver, European Association for the Study of Diabetes, European Association for the Study of Obesity, 2016; Chalasani et al., 2018; Eslam et al., 2020; Park et al., 2021). The burdens of these diseases and their sequelae are problematic in both developed and developing countries. Based on the results of modeling studies, NAFLD, non-alcoholic steatohepatitis (NASH), and liver fibrosis are projected to create very large clinical and economic burdens (Younossi et al., 2019; Tampi et al., 2020; Ito et al., 2021; Park et al., 2021; Phisalprapa et al., 2021). Slowing the incidence rates and preventing the sequelae of NAFLD are important solutions to reduce the burdens.

Behavioral modification, weight reduction, and drug therapies are hoped to be effective treatments for NAFLD. Despite the fact that there is no approved pharmacological treatment for NAFLD and non-biopsy-proven patients (European Association for the Study of the Liver, European Association for the Study of Diabetes, European Association for the Study of Obesity, 2016; Chalasani et al., 2018; Leoni et al., 2018), behavioral changes that lead to weight reduction, fat- and carbohydrate-diet adjustment, exercise, proper sleep, and coffee consumption have caught the attention of researchers (European Association for the Study of the Liver, European Association for the Study of Diabetes, European Association for the Study of Obesity; Chalasani et al., 2018; Leoni et al., 2018; Calabrò et al., 2020). Numerous studies have shown that coffee consumption has many positive effects on the liver, such as reducing the risk of fatty liver disease, lowering the severity of hepatic steatosis, and slowing the progression of fibrosis (Molloy et al., 2012; Zelber-Sagi et al., 2015). Recent meta-analyses and narrative reviews have reported many favorable clinical effects in people who regularly consume coffee or caffeine-containing products compared with those who do not (Calabrò et al., 2020)—for example, coffee consumption could be a protective factor against NAFLD and liver fibrosis (Marventano et al., 2016; Shen et al., 2016; Wijarnpreecha et al., 2017; Chen et al., 2019; Calabrò et al., 2020). Additionally, coffee extract improved the lipid profile and body mass index of patients with NAFLD (Hosseinabadi et al., 2020). Various mechanisms of NAFLD and liver fibrosis have been proposed, one of which is associated with transforming growth factor-beta (TGF-β) and oxidative stress (Tarantino et al., 2019). The hypotheses of the positive effects of coffee on the liver are that caffeine may help reduce TGF-β in liver cells and inhibit hepatic stellate cell activity (Shim et al., 2013). Additionally, non-caffeine substances, such as cafestol, kahweol, and chlorogenic acid, are antioxidants that may help reduce triglyceride and cholesterol deposition in hepatocytes and prevent cancer. Moreover, phenolic substances, including ferulic acid, have been introduced for the treatment of cardiovascular diseases and diabetes (Calabrò et al., 2020).

However, several studies have reported non-significant benefits of coffee on clinical and laboratory outcomes related to NAFLD (Calabrò et al., 2020). The effects of coffee consumption on the risk of NAFLD in the general population and liver fibrosis in patients with NAFLD are controversial. Therefore, we conducted this study using a reliable methodology—an umbrella review—to investigate the summarized effects of coffee consumption found by previous systematic reviews and meta-analyses. Additionally, we conducted a new systematic review and meta-analysis (SRMA) that included recently published primary studies to obtain up-to-date results with more power from a larger sample size.

## Methods

### Search Strategy and Study Selection

This study consisted of two parts: an umbrella review and a SRMA. Separate searches were carried out to obtain articles for each part of the study. PubMed, EMBASE, Cochrane, Scopus, and CINAHL databases were searched. There was no language restriction in our inclusion criteria for eligible studies. The searches were performed on September 9, 2021. This research project was conducted and reported according to the statement of Preferred Reporting Items for Systematic Reviews and Meta-analyses (PRISMA) (Liberati et al., 2009; Page et al., 2021). The study was registered with PROSPERO (registration number, CRD42021226607).

### Umbrella Review

The search terms for the umbrella review were (“coffee consumption” OR “caffeine consumption”) AND (“NAFLD” OR “non-alcoholic fatty liver disease”) AND (“systematic review” OR “meta-analysis”). The eligibility criteria were as follows:

1. A systematic review with/without meta-analysis
2. A meta-analysis that reported any pooled results of risk ratios (RRs) or odds ratios (ORs) by comparing exposures (did and did not drink coffee) with outcomes (incidence of NAFLD and/or liver fibrosis)
3. A meta-analysis that reported the mean differences (MDs) in the coffee consumption of the two groups of subjects with different hepatic outcomes
4. Research on NAFLD and liver fibrosis that had been diagnosed by reliable methods, such as elevated liver enzymes, ultrasonography, and/or liver biopsy

Publications that met the eligibility criteria were included in the umbrella review.

### Systematic Review and Meta-analysis

The search terms for the SRMA were (“coffee consumption” OR “caffeine consumption”) AND (“NAFLD” OR “non-alcoholic fatty liver disease” OR “NASH” OR “non-alcoholic steatohepatitis” OR “fatty liver”). The eligibility criteria were as follows:

1. A case–control, cohort, or cross-sectional design study
2. A study that compared liver outcomes (NAFLD incidence and/or liver fibrosis) of two groups of participants who did and did not drink coffee and reported the results as adjusted RRs or ORs with 95% confidence intervals (95% CI)
3. A study that reported the MDs with 95% CI in the coffee consumption of the two groups of subjects with different hepatic outcomes
4. Research on NAFLD and liver fibrosis that had been diagnosed using reliable methods, such as elevated liver enzymes, ultrasonography, and/or liver biopsy

Primary studies that met the eligibility criteria were included in the SRMA.

### Data Extraction and Quality Assessment

In the umbrella review, we extracted information from the included systematic reviews and meta-analyses following the Joanna Briggs Institute data extraction guide (Aromataris et al., 2015). The methodological quality of a meta-analysis was evaluated using A Measurement Tool to Assess Systematic Reviews (AMSTAR) 2 (Shea et al., 2017). If two or more studies were published in the same 24-month period for the same category of exposure and same outcomes, we selected the one with the highest AMSTAR 2 score for further analysis.

The quality of the primary studies in the SRMA was evaluated according to the Newcastle–Ottawa Quality Assessment Scale (NOS). Specifically, for the quality assessment of cross-sectional studies, the modified NOS described by Herzog et al. (2013) was used.

### Statistical Analysis

In the umbrella review, we comprehensively summarized the findings of previously published systematic reviews and meta-analyses. However, we did not review the full texts of the primary study articles examined by each meta-analysis.

For the SRMA, we used the DerSimonian and Laird random-effects model. The outcomes of interest were as follows:

1. An association between coffee consumption (1 to 2 and >2 cups/day *versus* <1 cup/day) and the incidence of NAFLD
2. An association between coffee consumption and reduction in liver fibrosis among patients with NAFLD
3. The difference in the consumption of coffee by the general population and by patients with NAFLD
4. The difference in coffee consumption of patients with NAFLD with no/mild and significant stages of liver fibrosis

The results are reported as pooled RRs and weighted mean differences (WMDs) along with 95% CIs. The *I* ^2^ statistic was calculated to determine the between-study heterogeneity. Publication bias was evaluated using Begg’s and Egger’s tests and by a visual investigation of funnel plots for potential asymmetry. All analyses were performed using Stata 14.1 (Stata Corp, College Station, TX, United States). A *p*-value of less than 0.05 was considered statistically significant.

## Results

### Umbrella Review

**FIGURE 1:**
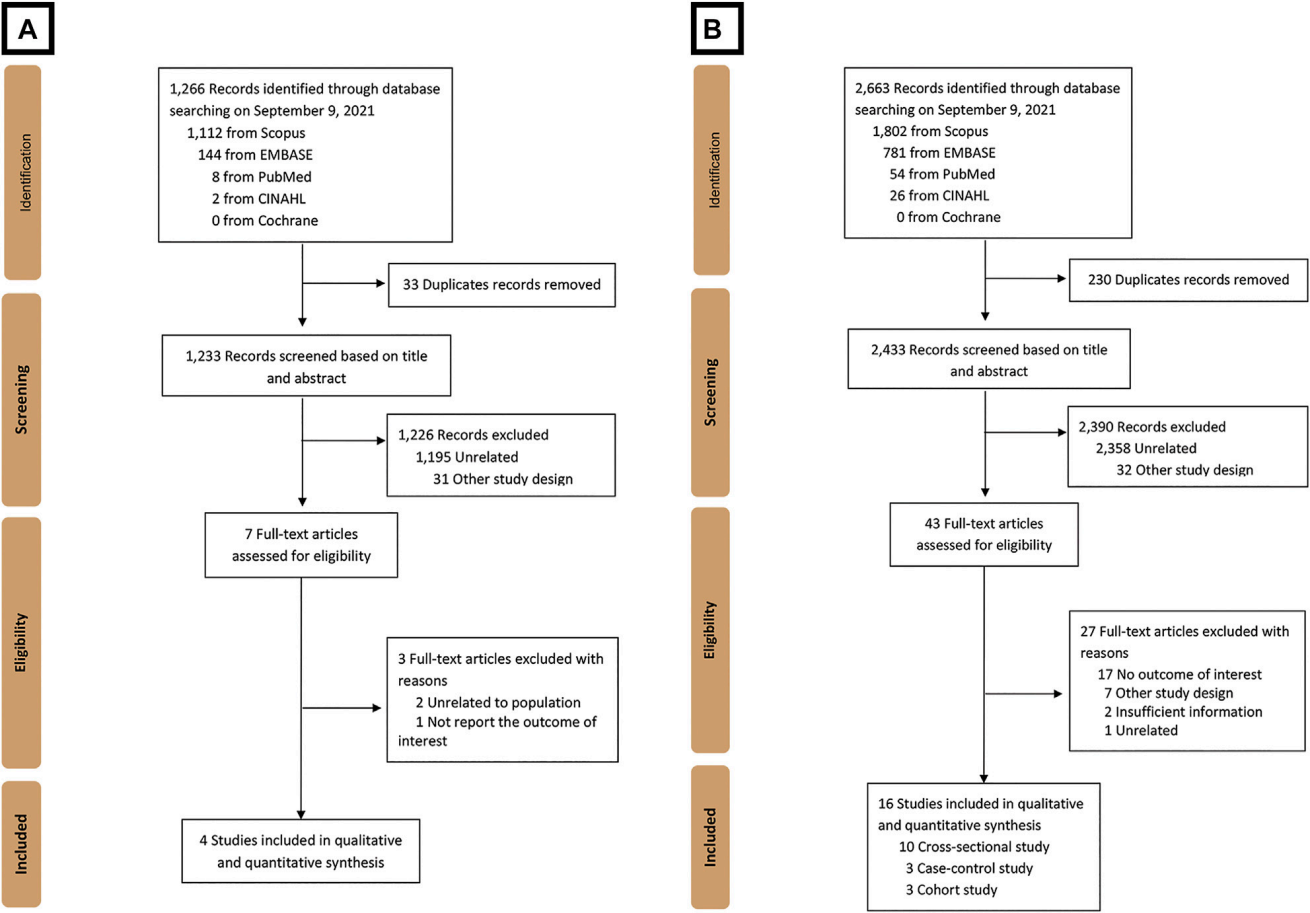
Flow diagram of the study identification, inclusion, and exclusion of **(A)** umbrella review and **(B)** systematic review and meta-analysis.

**FIGURE 2:**
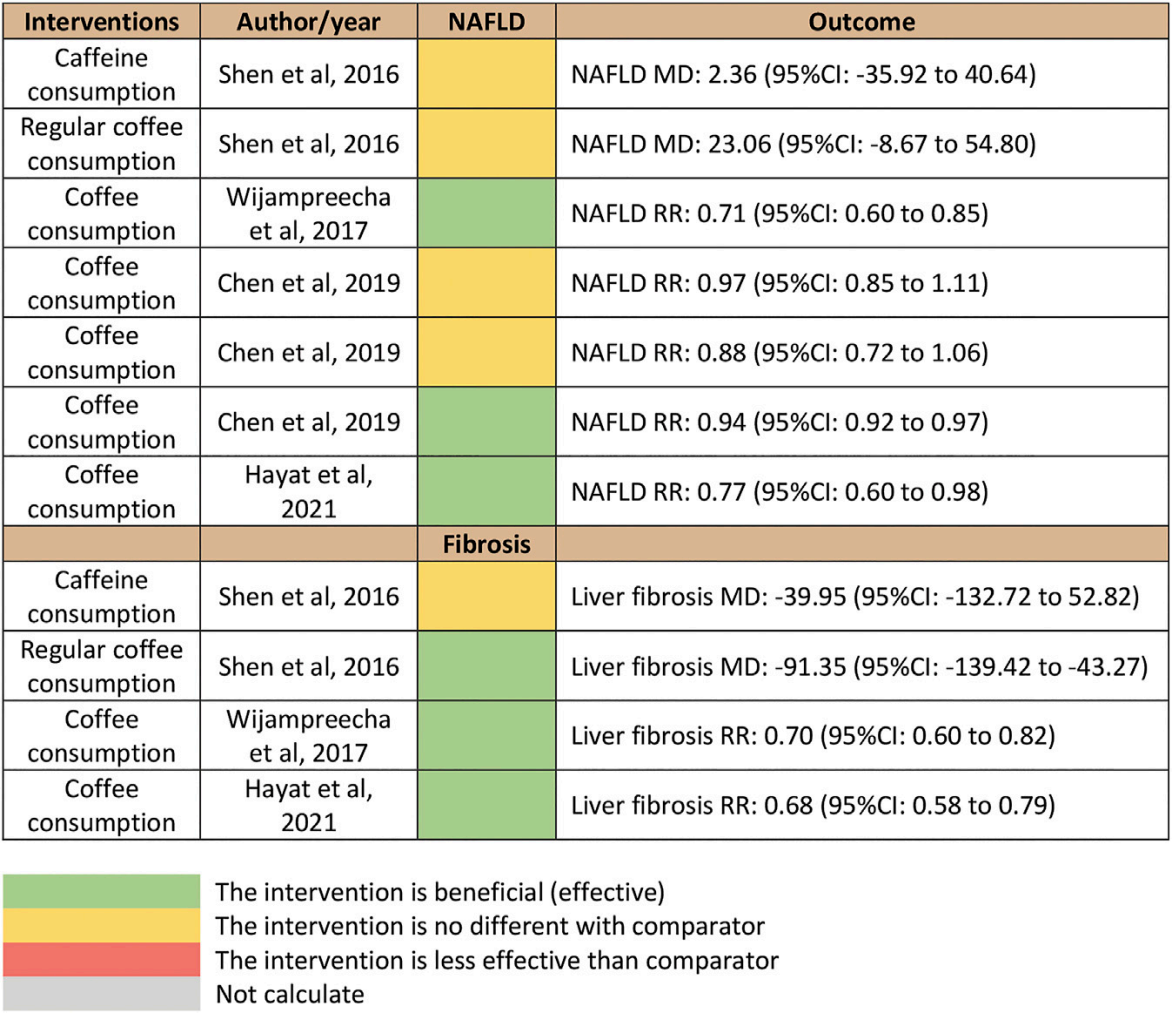
Summary of quantitative findings of umbrella review.

**FIGURE 3:**
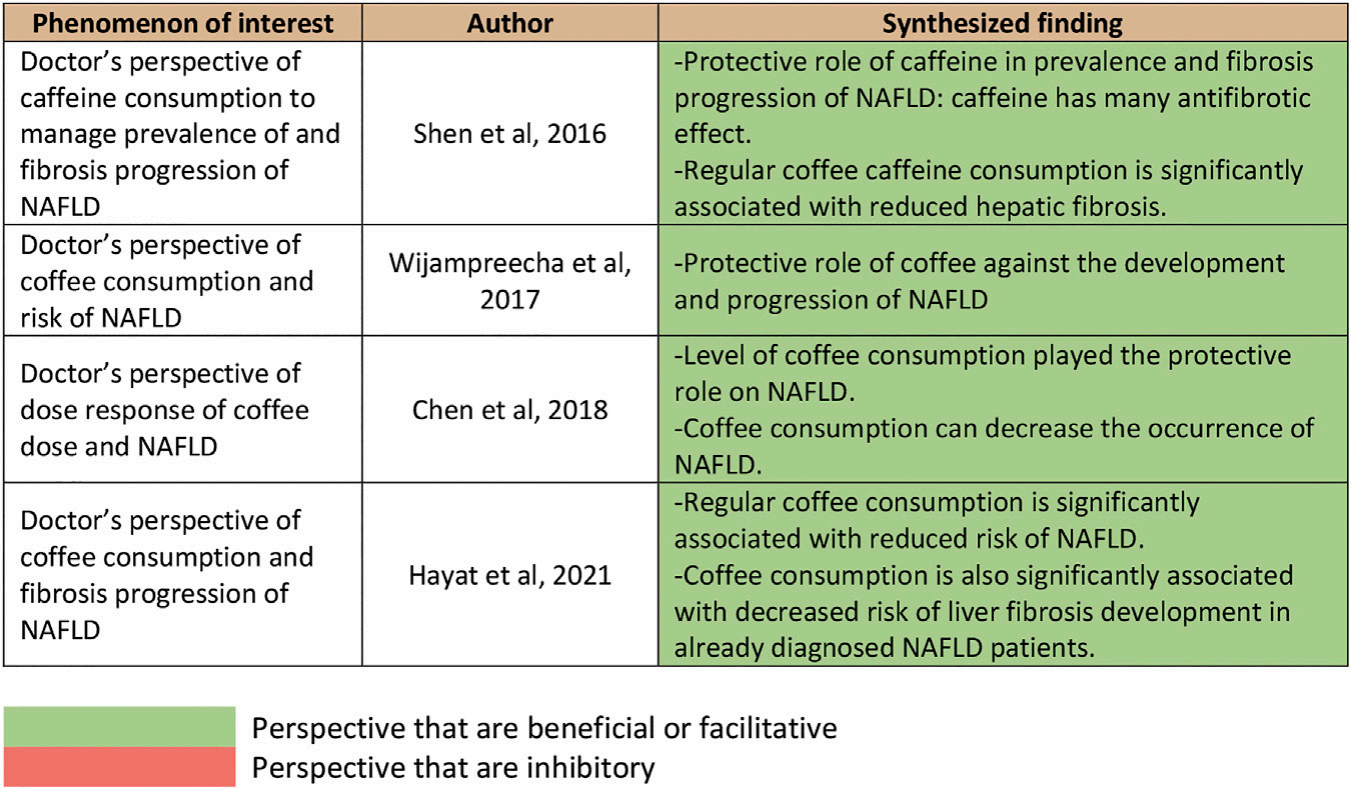
Summary of qualitative findings of umbrella review.

A total of 1,266 studies were retrieved using our search keywords. After the exclusion of 33 duplicate studies, the titles and abstracts of the remaining 1,233 studies were screened. This resulted in the elimination of 1,226 more studies (1,195 were not related and 31 used other study designs). Seven studies were available for full-length article review. Three of these were excluded because they did not report outcomes or have a study population within the scope of interest. The four studies (Shen et al., 2016; Wijarnpreecha et al., 2017; Chen et al., 2019; Hayat et al., 2021) that fully met the eligibility criteria were included in the umbrella review (Figure 1A). Their summarized quantitative and qualitative findings are presented in Figures 2, 3, respectively. They were considered high-quality reviews based on AMSTAR 2 criteria (Table 1). We did not test them for publication bias as the number of studies was too small.

**TABLE 1:** Quality assessment of studies included in the umbrella review.

AMSTAR 2	Summary			
	Shen *et al*. 2016	Wijarnpreecha *et al*. 2017	Chen *et al*. 2019	Hayat *et al*. 2021
1. Did the research questions and inclusion criteria for the review include the components of PICO?	Yes	Yes	Yes	Yes
2. Did the report of the review contain an explicit statement that the review methods were established prior to the conduct of the review and did the report justify any significant deviations from the protocol?	Yes	Yes	Yes	Yes
3. Did the review authors explain their selection of the study designs for inclusion in the review?	Yes	Yes	Yes	Yes
4. Did the review authors use a comprehensive literature search strategy?	Partial yes	Partial yes	Yes	Partial yes
5. Did the review authors perform study selection in duplicate?	Yes	Yes	Yes	Yes
6. Did the review authors perform data extraction in duplicate?	Yes	Yes	Yes	Yes
7. Did the review authors provide a list of excluded studies and justify the exclusions?	Yes	Yes	Yes	Yes
8. Did the review authors describe the included studies in adequate detail?	Yes	Yes	Yes	Yes
9. Did the review authors use a satisfactory technique for assessing the risk of bias (RoB) in individual studies that were included in the review?	Yes	Yes	Yes	Yes
10. Did the review authors report on the sources of funding for the studies included in the review?	Yes	Yes	Yes	Yes
11. If meta-analysis was performed, did the review authors use appropriate methods for statistical combination of results?	Yes	Yes	Yes	Yes
12. If meta-analysis was performed, did the review authors assess the potential impact of RoB in individual studies on the results of the meta-analysis or other evidence synthesis?	Yes	Yes	Yes	Yes
13. Did the review authors account for RoB in individual studies when interpreting/discussing the results of the review?	Yes	Yes	Yes	Yes
14. Did the review authors provide a satisfactory explanation for and discussion of any heterogeneity observed in the results of the review?	Yes	Yes	Yes	Yes
15. If they performed quantitative synthesis, did the review authors carry out an adequate investigation of publication bias (small study bias) and discuss its likely impact on the results of the review?	Yes	Yes	Yes	Yes
16. Did the review authors report any potential sources of conflict of interest, including any funding they received for conducting the review?	Yes	Yes	Yes	Yes
Result	High-quality review	High-quality review	High-quality review	High-quality review

### Coffee Consumption and Incidence of NAFLD in the General Population

Quantitative analyses from three studies by Wijarnpreecha et al. (2017), Chen et al. (2019), and Hayat et al. (2021) provided significant association outcomes between coffee consumption and incidence of NAFLD, with pooled RRs of 0.71 (95% CI, 0.60–0.85), 0.94 (95% CI, 0.92–0.97), and 0.77 (95% CI, 0.60–0.98), respectively. In contrast, the study by Shen et al. (2016) reported a non-significant outcome for daily coffee consumption, with an MD of 23.06 mg/day (95% CI, −8.67–54.80). The results are illustrated in Figure 2. The qualitative outcomes included the perspective of doctors that coffee consumption provided a beneficial protective effect on NAFLD among the general population (Figure 3).

### Coffee Consumption and Liver Fibrosis in Patients With NAFLD

Quantitative outcomes from three studies by Wijarnpreecha *et al*. (2017), Shen et al. (2016), and Hayat et al. (2021) indicated that there was a significant association between coffee consumption and liver fibrosis in patients with NAFLD, with a pooled RR of 0.70 (95% CI, 0.60–0.82), MD of −91.35 mg/day (95% CI, −139.42–−43.27), and RR of 0.68 (95% CI, 0.58–0.79; Figure 2). The qualitative outcomes included the perspective of doctors that coffee was beneficial to the fibrosis outcomes of patients with NAFLD (Figure 3).

### Systematic Review and Meta-analysis

**FIGURE 4:**
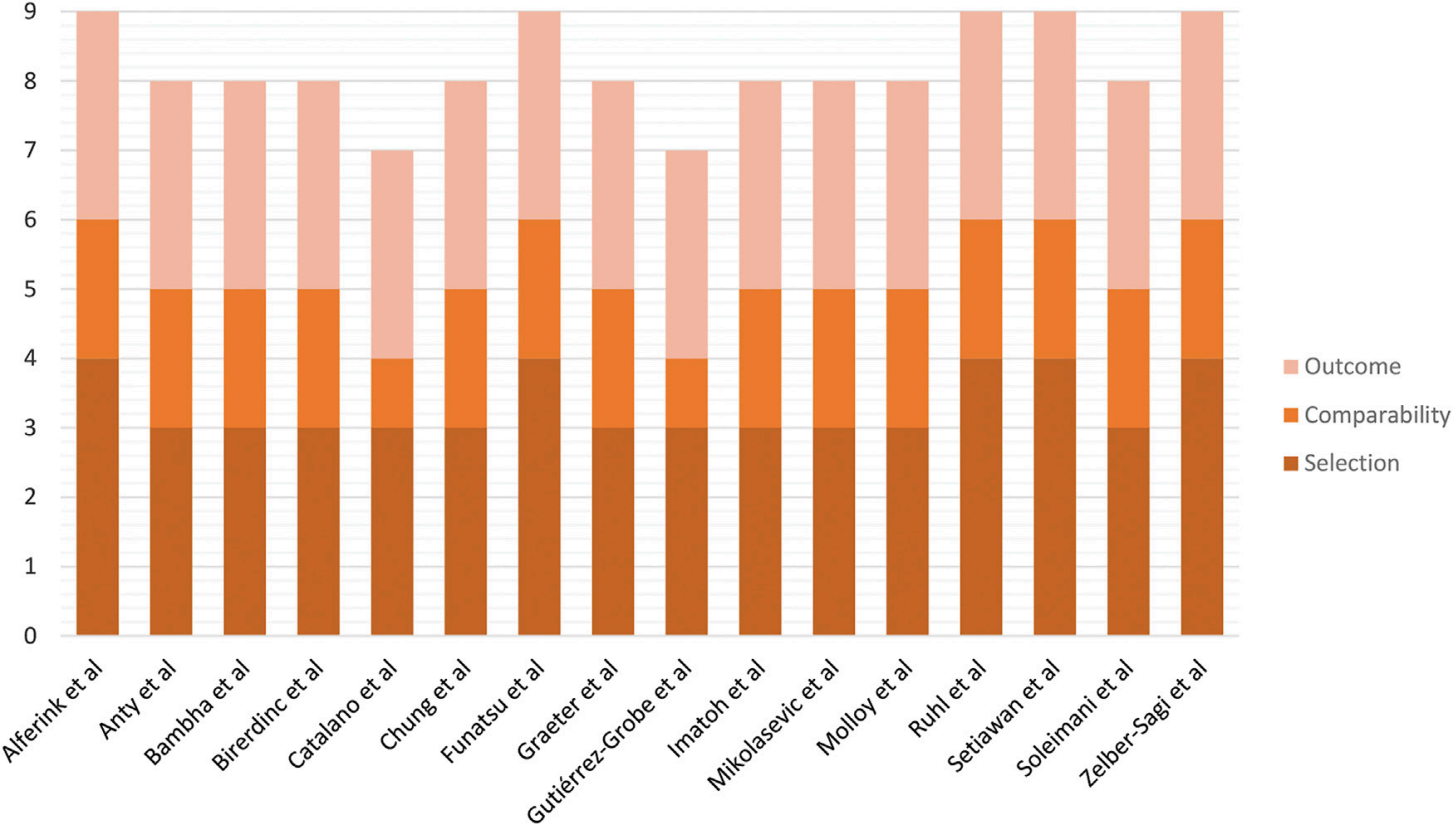
Risk of bias assessment of cohort studies included in the meta-analysis using the Newcastle–Ottawa Scale.

A total of 2,663 studies were retrieved using our search keywords. After excluding duplicate records, the titles and abstracts of 2,433 studies were screened. Of these, 2,390 were excluded due to irrelevance, leaving 43 for full-length article review. Twenty-seven of these were subsequently excluded because they did not meet our eligibility criteria. Eventually, 16 studies (*n* = 171,096) were available for data analysis (Figure 1B) (Ruhl and Everhart, 2005; Catalano et al., 2010; Funatsu et al., 2011; Anty et al., 2012; Birerdinc et al., 2012; Gutiérrez-Grobe et al., 2012; Molloy et al., 2012; Bambha et al., 2014; Graeter et al., 2015; Imatoh et al., 2015; Zelber-Sagi et al., 2015; Alferink et al., 2017; Setiawan et al., 2017; Soleimani et al., 2019; Chung et al., 2020; Mikolasevic et al., 2021). The characteristics of the included studies were extracted (Table 2). Quality evaluations using NOS indicated that all were high-quality studies (Figure 4).

**TABLE 2:** Characteristics of the studies included in the systematic review and meta-analysis.

No	Author, year	Country	Study design	Detail of participants	Sample size	Exposure category	Exposure measurement	Outcome	Outcome ascertainment	Confounder adjustment
1	Alferink LJM, 2017 (Alferink et al., 2017)	Netherlands	Cross-sectional study	Population-based cohort of participants aged 45 years and older who visited the research center between January 2011 and September 2013	2,258	0, 1–2, ≥3 cups/day	Questionnaire	HS and SF	US	Energy intake, BMI, gender, age, steatosis, ALT, excessive alcohol intake, current or former smoking and HOMA-IR, soda consumption, cream and sugar use, DHDI, and physical activity
2	Anty R, 2012 (Anty et al., 2012)	France	Cross-sectional study	Patients with consecutive severe and morbid obesity and who were referred for bariatric surgery between December 2009 and July 2011	195	NA	Interview and questionnaire	NAFLD and SF	Liver biopsy	AST, HOMA-IR, metabolic syndrome, NASH
3	Bambha K, 2014 (Bambha et al., 2014)	United States	Cross-sectional study	Participants enrolled in the United States multicenter collaborative research consortium of the NASH Clinical Research Network (NASH CRN) from 2004 to 2008	782	0, <0, <1, 1–2, ≥2 cups/day	Questionnaire	SF	Liver biopsy	Age, gender, ethnicity, waist circumference, AST, GGT, diabetes, smoking, alcohol, biopsy length, HOMA-IR
4	Birerdinc A, 2011 (Birerdinc et al., 2012)	United States	Cross-sectional study	Participants were obtained from four continuous cycles of National Health and Nutrition Examination Survey (NHANES) between 2001 and 2008	18,550	NA	Questionnaires	NAFLD	Elevated serum AST, ALT	Age, gender, ethnicity, metabolic syndrome components
5	Catalano D, 2010 (Catalano et al., 2010)	Italy	Case-control study	Total NAFLD consecutive patients were studied in gastroenterology and nutrition unit operating as an autonomous outpatient clinic and day hospital	310	0,1–2, ≥3 cups/day	Questionnaires	NAFLD	US (BLS score)	NA
6	Chung HK, 2020 (Chung et al., 2020)	Korea	Cohort study	Participants who collaborated in a comprehensive health screening program at least twice at Kangbuk Samsung Hospital from 2011 to 2016	91,436	Coffee intake (cups/day)	Questionnaire	NAFLD	US	Age, sex, education, exercise, smoking, alcohol intake, center, year, BMI, total energy intake, triglyceride, LDL, HDL, glucose, AST, ALT, change of alcohol, change of BMI, change of exercise
7	Funatsu K, 2011 (Funatsu et al., 2011)	Japan	Case–control study	Male office workers employed at a single company who were between the ages of 25 and 60 years in 1999 were recruited	1,612	Coffee intake (cups/day)	Questionnaire	NAFLD	US	Age, BMI, exercise level, daily coffee intake, daily alcohol intake
8	Graeter T, 2015 (Graeter et al., 2015)	Germany	Cross-sectional study	Participants who registered inhabitants between the ages of 10 and 65 years old received invitations by mail to participate in a random population-based sample	1,452	>1 and 1 cap/day, <1 cup/week, <1 cup/month, seldom/rarely	Questionnaire	HS	ATL, US	Age, gender, BMI
9	Grobe YG, 2012 (Gutiérrez-Grobe et al., 2012)	Mexico	Case–control study	Population selected from a consecutive series of asymptomatic subjects who were referred to the checkup unit by their companies as an annual employment requirement	130	Caffeine (mg/day)	Questionnaire	NAFLD	US	NA
10	Imatoh T, 2015 (Imatoh et al., 2015)	Japan	Cross-sectional study	Male office workers who received annual health checkups at the clinic located in the center of Fukuoka city recruited from March 2010 to November 2010	1,030	0, 1–2, ≥3 cups/day	Questionnaire	HS	US	Age, BMI, smoking status, alcohol drinking status, green tea consumption
11	Mikolasevic I, 2020 (Mikolasevic et al., 2021)	Croatia	Cross-sectional study	All patients visiting the Gastroenterology Department of Clinical Hospital Centre Rijeka between April 2013 and May 2019	1,998	0, 1–2, ≥3 cups/day	Questionnaire	NAFLD	US	Age, sex, BMI, waist circumference, hypertension, diabetes mellitus, ALT, GGT
12	Molloy JW, 2012 (Molloy et al., 2012)	United States	Cross-sectional study	Patients were identified from medical records of the Brooke Army Medical Center hepatology clinic	306	NA	Questionnaire	NASH, Steatosis, Fibrosis	Liver biopsy, NASH score	NA
13	Ruhl CE, 2005 (Ruhl and Everhart, 2005)	United States	Cohort study	Patients from the Third National Health and Nutrition Examination Survey (NHANES III) which was conducted in the United States from 1988 to 1994 by the National Center	5,944	0, <1, 1–2, >2 cups/day	Interview, examination, laboratory	NAFLD	Serum ALT	Age, sex, ethnicity, cigarette smoking
14	Setiawan VW, 2017 (Setiawan et al., 2017)	United States	Cohort study	Participants who were enrolled in the Medicare fee-for-service and completed questionnaires on coffee intake and confounders whose age was 45–75 years at enrollment between 1993 and 1996	44,576	Never <1, 1, 2–3, ≥4 cups/day	Questionnaire	NAFLD	US	Education, BMI, diabetes, smoking status, alcohol intake
15	Soleimani D, 2019 (Soleimani et al., 2019)	Iran	Cross-sectional study	Adult patients 20–60 years were randomly recruited from the consecutive gastroenterology outpatient clinics	170	0–3 cup of coffee consumption per day	Diet information was collected by using a 3-days dietary record	Liver fibrosis	FibroScan	Age, gender, BMI, education, smoking, diabetes, antidiabetic medication use, dietary supplement, physical activity, energy intake
16	Zelber-Sagi S, 2014 (Zelber-Sagi et al., 2015)	Israel	Cross-sectional study	Patients who were located and agreed to participate before the application of exclusion criteria	347	<3 and ≥3 cups/day	Questionnaire	NASH, Steatosis, Fibrosis	US, FibroTest, SteatoTest, NashTest	Smoking, sugar, and physical activity (minute/weeks), serum cholesterol levels, dietary fat, calorie intake

ALT, alanine aminotransferase; AST, aspartate aminotransferase; BLS, bright liver score; BMI, body mass index; DHDI, Dutch Healthy Diet Index; GGT, gamma-glutamyl transferase; HOMA-IR, homeostatic model assessment—insulin resistance; HS, hepatic steatosis; NA, not applicable; NAFLD, non-alcoholic fatty liver disease; NASH, non-alcoholic steatohepatitis; SF, significant fibrosis; US, ultrasonography.

### Coffee Consumption and Incidence of NAFLD in the General Population

**FIGURE 5:**
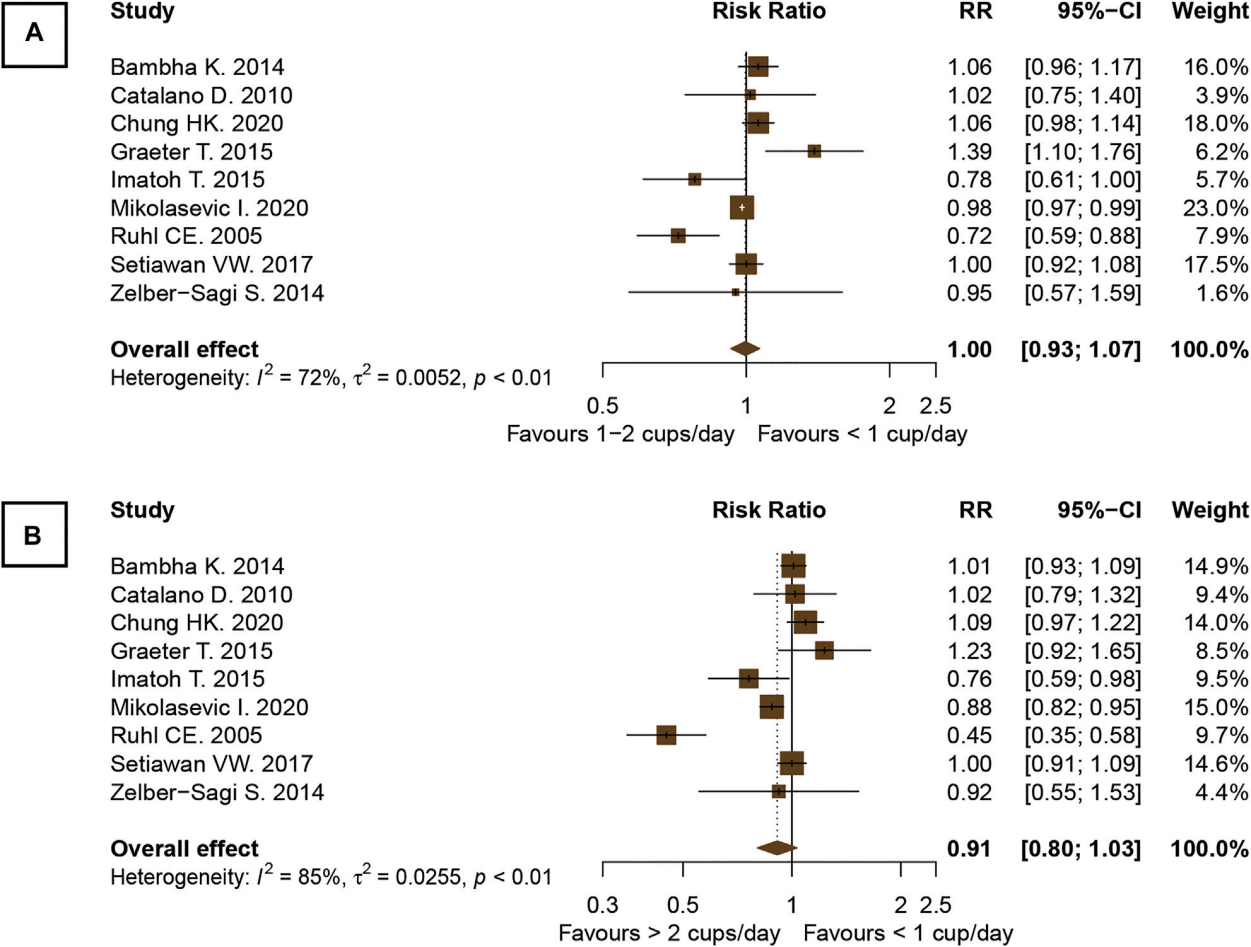
Forest plots of the association of coffee consumption and the incidence of NAFLD in the general population: **(A)** 1 to 2 cups of coffee per day *versus* <1 cup per day and **(B)** >2 cups per day *versus* <1 cup per day.

The pooled RR of nine observational studies (*n* = 147,875) did not show a significant difference in the incidence of NAFLD of the population who drank one or more cups of coffee per day and <1 cup/day *versus* those who did not (Ruhl and Everhart, 2005; Catalano et al., 2010; Bambha et al., 2014; Graeter et al., 2015; Imatoh et al., 2015; Zelber-Sagi et al., 2015; Setiawan et al., 2017; Chung et al., 2020; Mikolasevic et al., 2021). The analyses revealed that neither the consumption of one to two cups of coffee/day nor the consumption of even more than two cups/day was associated with a reduction in the incidence of NAFLD in the general population compared with the population who drank less than one cup of coffee per day (RR, 1.00; 95% CI, 0.93–1.07; *I* ^2^, 72% and RR, 0.91; 95% CI, 0.80–1.03; *I* ^*2*^, 85%, respectively; Figure 5).

### Coffee Consumption and Liver Fibrosis in Patients With NAFLD

**FIGURE 6:**
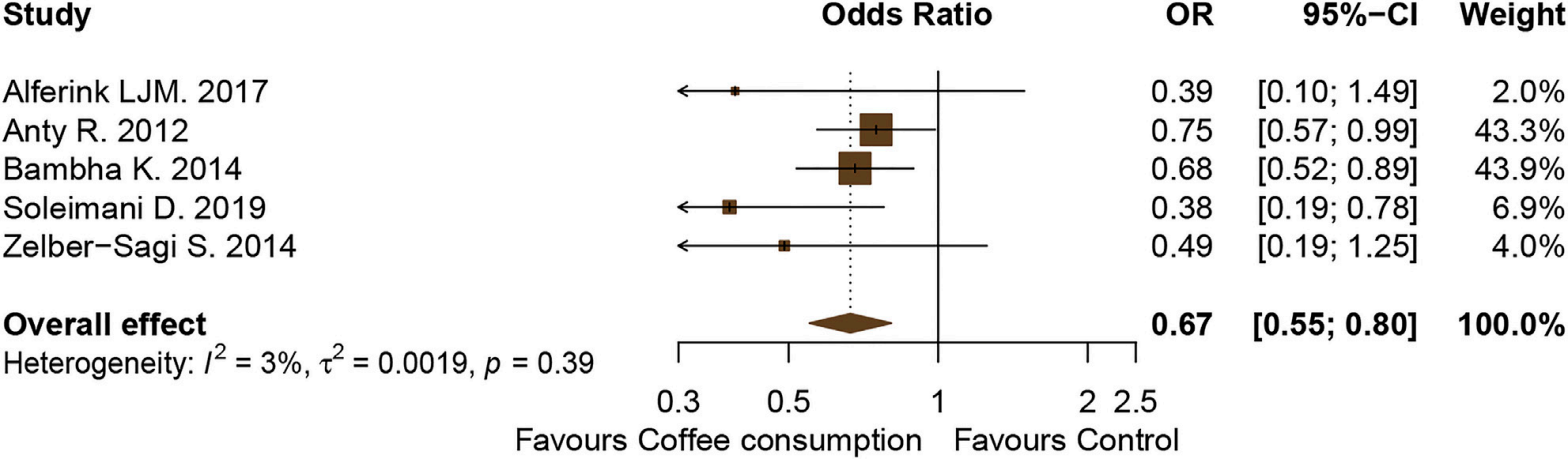
Forest plot of the association of coffee consumption and the liver fibrosis outcomes of patients with NAFLD who drank coffee *versus* those who did not drink coffee (control).

A total of five studies (*n* = 2,948) were included in this outcome analysis (Anty et al., 2012; Bambha et al., 2014; Zelber-Sagi et al., 2015; Alferink et al., 2017; Soleimani et al., 2019). The results showed that coffee consumption was significantly associated with a reduction in hepatic fibrosis in patients with NAFLD (OR, 0.67; 95% CI, 0.55–0.80; *I* ^2^, 3%; Figure 6).

### Coffee Consumption of the General Population and Patients With NAFLD

Four observational studies explored the association between coffee consumption by the general population and by patients with NAFLD (Catalano et al., 2010; Funatsu et al., 2011; Birerdinc et al., 2012; Gutiérrez-Grobe et al., 2012). There was a total of 19,482 individuals (general population, 17,322; NAFLD patients, 2,160). The pooled result showed that the general population did not consume more coffee than people with NAFLD (WMD, −17.11 mg/day; 95% CI, −51.41–17.20; *I* ^2^, 85%), as illustrated in Supplementary Figure S1.

### Coffee Consumption of NAFLD Patients With No/Mild and Significant Stages of Liver Fibrosis

Four observational studies investigated the difference in the coffee consumption of NAFLD patients with no/mild and significant stages of liver fibrosis (Anty et al., 2012; Molloy et al., 2012; Bambha et al., 2014; Alferink et al., 2017). Of the 3,331 patients, 2,912 had no/mild fibrosis, while 419 had significant fibrosis. The pooled result showed that there was no difference in the coffee consumption of the two groups of patients with different stages of fibrosis (WMD, −33.78 mg/day; 95% CI, −90.26–22.71; *I* ^2^, 81%). The comparison is illustrated in Supplementary Figure S2.

### Publication Bias

**FIGURE 7:**
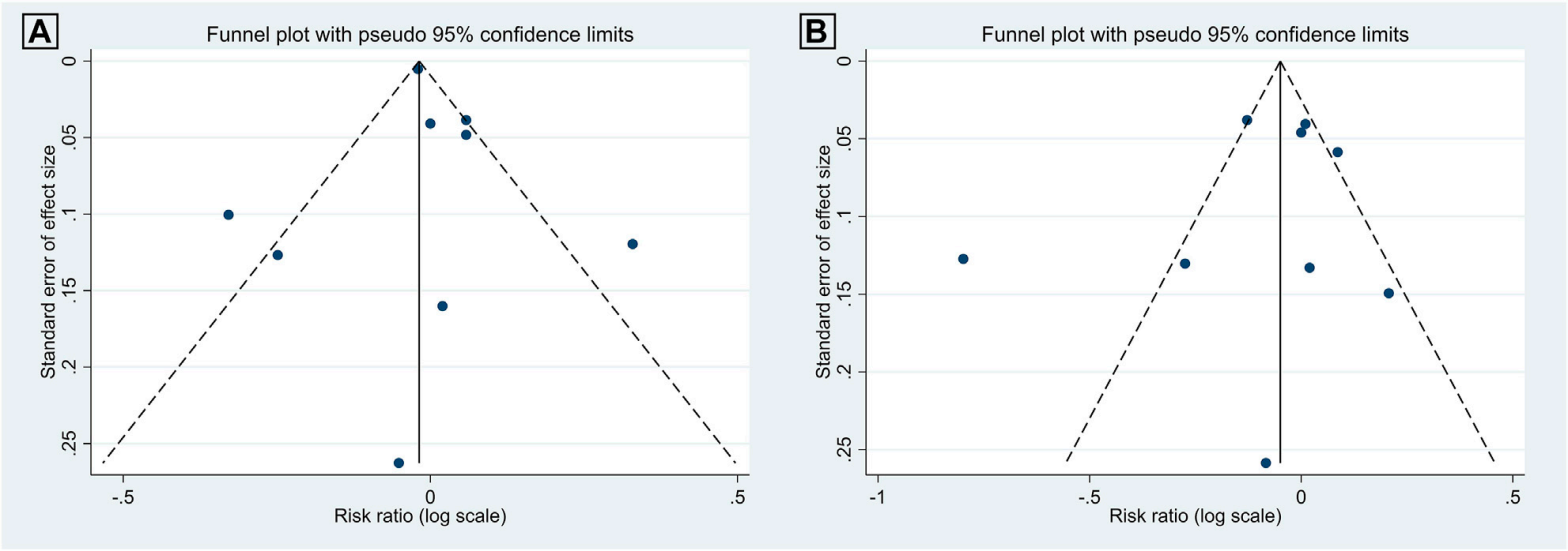
Funnel plots of the studies investigating the effects of coffee consumption on the incidence of NAFLD in the general population: **(A)** 1 to 2 cups/day *versus* <1 cup/day and **(B)** two cups/day *versus* <1 cup/day.

**FIGURE 8:**
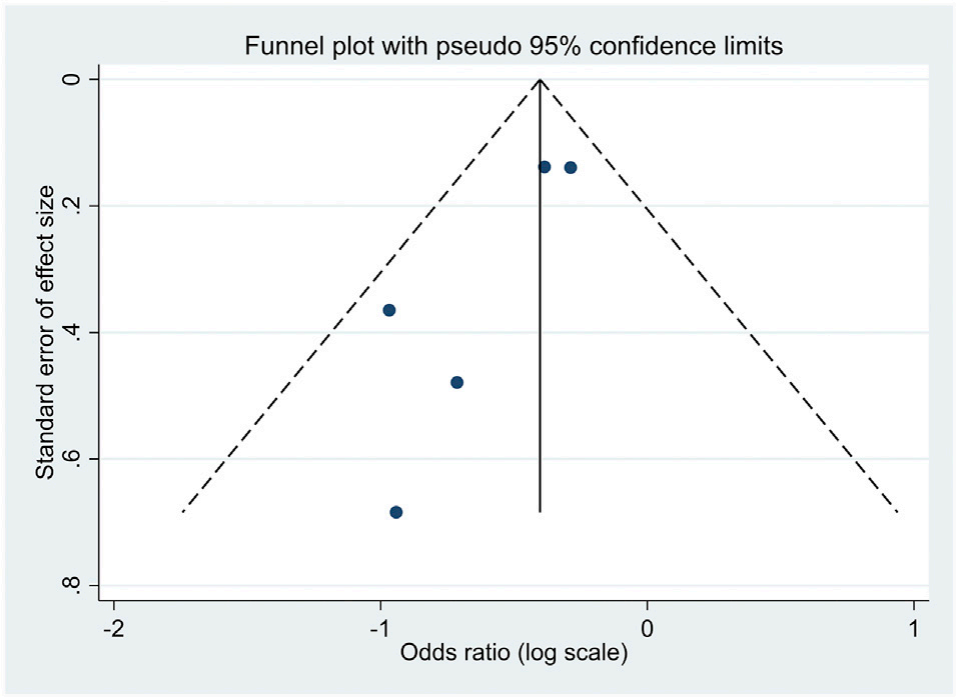
Funnel plot of the studies investigating the effects of coffee consumption and liver fibrosis in patients with NAFLD.

There was no evidence of publication bias among the included studies, as illustrated in Figures 7, 8. For articles comparing the association of coffee consumption of one to two cups/day and of <1 cup/day with the incidence of NAFLD in the general population, the *p*-values were 0.677 for Begg’s test and 0.747 for Egger’s test. Regarding the association of coffee consumption of >2 cups/day and of <1 cup/day with the incidence of NAFLD in the general population, the *p*-value of Begg’s and Egger’s tests was 0.835 and 0.533, respectively. Furthermore, there was no publication bias among the studies investigating the association of coffee consumption with liver fibrosis in patients with NAFLD. The *p*-value for Begg’s and Egger’s tests was 0.327 and 0.060, respectively.

## Discussion

Both the qualitative and quantitative outcomes of the umbrella review showed that most of the previous systematic reviews and meta-analyses reported an association between coffee consumption and a reduction in the incidence of NAFLD in the general population. Moreover, the results showed that, from the point of view of doctors, coffee consumption had the beneficial effect of reducing the degree of liver fibrosis among patients with NAFLD.

Many studies have reported the hepatoprotective effect of coffee. This may be the result of several caffeine and non-caffeine substances (Shen et al., 2016; Wijarnpreecha et al., 2017; Chen et al., 2019; Hayat et al., 2021). Caffeine was shown to reduce oxidative stress and liver inflammation in an *in vitro* study. Moreover, its mechanisms of action showed signs of anti-fibrotic properties in hepatocytes (Gressner et al., 2008). Preclinical research found that other non-caffeine compounds also showed antioxidative characteristics that could reduce inflammatory reactions (Furtado et al., 2012). In addition to anti-inflammatory effects, antioxidants also have a significant role in reducing the accumulation of lipids in liver cells (Calabrò et al., 2020). The outcomes of the included systematic reviews and meta-analyses were particularly evident in their clinical aspects. They established that consuming coffee provided the benefits of reducing both the risk of NAFLD in the general population and the severity of fibrosis in patients with NAFLD (Shen et al., 2016; Wijarnpreecha et al., 2017; Chen et al., 2019; Hayat et al., 2021).

However, in the present SRMA, which included a much larger number of samples, we found that coffee consumption was not associated with a lower incidence of NAFLD in the general population. This non-significant outcome may be due to many reasons—for example, there were heterogeneities between the primary studies in terms of their methodologies, the differences in the types and amounts of coffee consumed, and the various tools used to investigate the basis of coffee consumption in the subjects. Thus, we were unable to confirm the protective effect of coffee against NAFLD in the general population. Even individuals who consumed more than two cups of coffee daily did not show a lower risk of NAFLD than those who drank less than one cup per day. However, among patients with NAFLD, coffee consumption was significantly associated with a reduction in hepatic fibrosis. On the other hand, the pooled results of four studies (Anty et al., 2012; Molloy et al., 2012; Bambha et al., 2014; Alferink et al., 2017) with a total of 3,331 patients with NAFLD indicated that there was no difference between the coffee intake of patients with no/mild liver fibrosis and those with significant liver fibrosis. Thus, more research is needed on the coffee intake required to reduce the severity of liver fibrosis in patients with NAFLD.

The benefits of coffee for patients with NAFLD can be explained by the underlying mechanism of NAFLD. Since coffee is composed of many substances, those that offer any effect that intervenes in the etiology of NAFLD should result in favorable clinical outcomes. As mentioned above, both caffeine and non-caffeine substances were preliminarily proven in preclinical studies to be able to block pathways associated with oxidative stress and TGF-β. These pathways happen to be one of the mechanisms behind the incidence of NAFLD and liver fibrosis (Tarantino et al., 2019). Still, various factors could alter clinical results in the real world. The inconsistent findings of our study were not unexpected due to the number of undetermined confounders and variations among the included clinical studies.

The strengths of our study were that we not only evaluated and summarized previously published SRMAs on the topics mentioned above but also rigorously performed a new SRMA to determine the effects of coffee consumption. The umbrella review and SRMA were conducted following standard guidelines. The searches were performed using various reliable databases and were assessed without language restriction. The SRMAs in the umbrella review were considered high-quality reviews according to AMSTAR 2 criteria. As far as we are concerned, our SRMA encompasses the most up-to-date evidence available and measures the effects of coffee consumption using the largest sample size currently available. Despite conflicting results on the protective effects of NAFLD in the general population, the benefits of coffee consumption on liver fibrosis were identified in patients with NAFLD. The results of this study may encourage researchers to develop well-designed studies to confirm the hepatoprotective effects of coffee, which should be useful in clinical practice.

This study had several limitations. First, it consisted of observational studies that could only show an association—not a causal relationship—between coffee consumption and liver outcomes. Second, the methodologies of the included studies were quite heterogeneous. Remarkable heterogeneity among the studies was also shown through the *I* ^2^ statistic. Therefore, this current investigation adopted a random-effects model to pool the outcomes for the meta-analysis. Several of the included studies diagnosed NAFLD based on ultrasonography without confirmation by liver biopsy. Furthermore, the measurement of coffee intake in the primary articles was derived using a questionnaire at a single time point. This may not have provided an accurate assessment of the general coffee intake. The studies also used various means to measure coffee consumption, and some might have been prone to recall bias. Third, relevant information on coffee consumption, which may have influenced the outcomes of the present work, was not provided in the primary articles. This included details of the type of coffee, the components of the coffee, the brewing method, the amount of coffee per cup, and the drinking time. Thus, we were unable to analyze alterations in results when these factors changed. As an example, adding milk or sugar to coffee may increase fat accumulation in the liver, resulting in more unfavorable outcomes. Fourth, confounders of the included population were not used to adjust the outcomes in our analyses. The confounders were, for example, comorbidities, smoking, alcohol intake, physical activity, diet, and socioeconomic status.

In conclusion, our umbrella review and SRMA showed contrasting results on the benefits of coffee consumption on liver outcomes among the general population. Whether the consumption of coffee can be considered a preventive measure against NAFLD requires further investigation with a more rigid methodology. However, the results of both the umbrella review and the SRMA showed that the benefits of coffee consumption on liver fibrosis were significant for patients with NAFLD.

## Data Availability

The original contributions presented in the study are included in the article/Supplementary Material, and further inquiries can be directed to the corresponding authors.

## Abbreviations

AMSTAR, A MeaSurement Tool to Assess systematic Reviews; BMI, body mass index; CI, confidence interval; JBI, Joanna Briggs Institute; MD, mean difference; NAFLD, non-alcoholic fatty liver disease; NASH, non-alcoholic steatohepatitis; NOS, Newcastle–Ottawa quality assessment scale; OR, odds ratio; PRISMA, Preferred Reporting Items for Systematic Reviews and Meta-analyses; RR, risk ratio; SRMA, systematic review and meta-analysis; WMD, weighted mean difference.
