# Supplementary material for: Coffee Consumption and Non-alcoholic Fatty Liver Disease: An Umbrella Review and a Systematic Review and Meta-analysis
Source: Front Pharmacol. 2021 Dec 13;12:786596. doi: 10.3389/fphar.2021.786596 (PMC8710778; doi:10.3389/fphar.2021.786596)
Supplement: Supplementary file 1 [file DataSheet1.PDF]

Frontiers in Pharmacology

ELECTRONIC SUPPLEMENTARY MATERIALS

Coffee consumption and non-alcoholic fatty liver disease: an umbrella review, and systematic review and meta-analysis

Running title: Coffee consumption and NAFLD

Chayanis Kositamongkol[1], Sukrit Kanchanasurakit[2–6], Chiraphong Auttamalang[3], Nutkamon Inchai[3], Thanatchaporn Kabkaew[3], Sarunporn Kitpark[3], Nathorn Chaiyakunapruk[7], Acharaporn Duangjai[8], Surasak Saokaew[3–6,9–10,*], and Pochamana Phisalprapa[1,*]

[1] Division of Ambulatory Medicine, Department of Medicine, Faculty of Medicine Siriraj Hospital, Mahidol University, Bangkok, Thailand.
[2] Division of Pharmaceutical Care, Department of Pharmacy, Phrae Hospital, Phrae, Thailand.
[3] Division of Pharmacy Practice, Department of Pharmaceutical Care, School of Pharmaceutical Sciences, University of Phayao, Phayao, Thailand.
[4] Center of Health Outcomes Research and Therapeutic Safety (Cohorts), School of Pharmaceutical Sciences, University of Phayao, Phayao, Thailand
[5] Unit of Excellence on Clinical Outcomes Research and IntegratioN (UNICORN), School of Pharmaceutical Sciences, University of Phayao, Phayao, Thailand
[6] Unit of Excellence on Herbal Medicine, School of Pharmaceutical Sciences, University of Phayao, Phayao, Thailand
[7] Department of Pharmacotherapy, College of Pharmacy, University of Utah, Salt Lake City, Utah, USA
[8] Department of Physiology, School of Medical Sciences, University of Phayao, Phayao, Thailand
[9] Biofunctional Molecule Exploratory Research Group, Biomedicine Research Advancement Centre, School of Pharmacy, Monash University Malaysia, Bandar Sunway, Selangor Darul Ehsan, Malaysia
[10] Novel Bacteria and Drug Discovery Research Group, Microbiome and Bioresource Research Strength, Jeffrey Cheah School of Medicine and Health Sciences, Monash University Malaysia, Bandar Sunway, Selangor Darul Ehsan, Malaysia

[*] **Correspondence:**
Associate Professor Surasak Saokaew, B.Pharm., Pharm.D., B.PHCP., F.A.C.P., Ph.D.
Center of Health Outcomes Research and Therapeutic Safety (Cohorts), School of Pharmaceutical Sciences, University of Phayao, Maeka, Muang, Phayao, Thailand, 56000
Tel: +66 54 466 666 ext. 3204; Fax: +66 54 466 661; Email: surasak.sa@up.ac.th

Associate Professor Pochamana Phisalprapa, M.D., Ph.D.
Division of Ambulatory Medicine, Department of Medicine, Faculty of Medicine Siriraj Hospital, Mahidol University, Bangkok, Thailand, 10700
Tel: +66 2 419 7190; Fax: +66 2 419 7190; E-mail: coco_a105@hotmail.com

TABLE OF CONTENTS

Appendix 1: Results of the systematic review and meta-analysis 3

Supplementary Figure S1 Forest plot indicated coffee consumption (mg/day) of the general population (control) and patients with NAFLD..3

Supplementary Figure S2 Forest plot of coffee consumption (mg/day) of NAFLD patients with no/mild and significant stages of liver fibrosis 3

Appendix 1: Results of the systematic review and meta-analysis

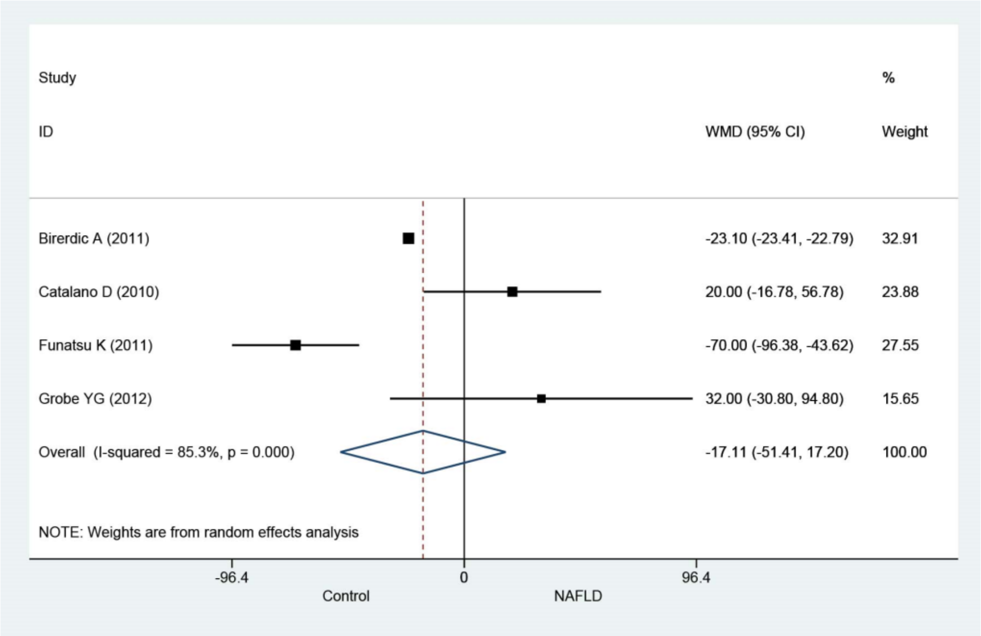

WMD, weighted mean difference

Supplementary Figure S1 Forest plot indicated coffee consumption (mg/day) of the general population (control) and patients with NAFLD

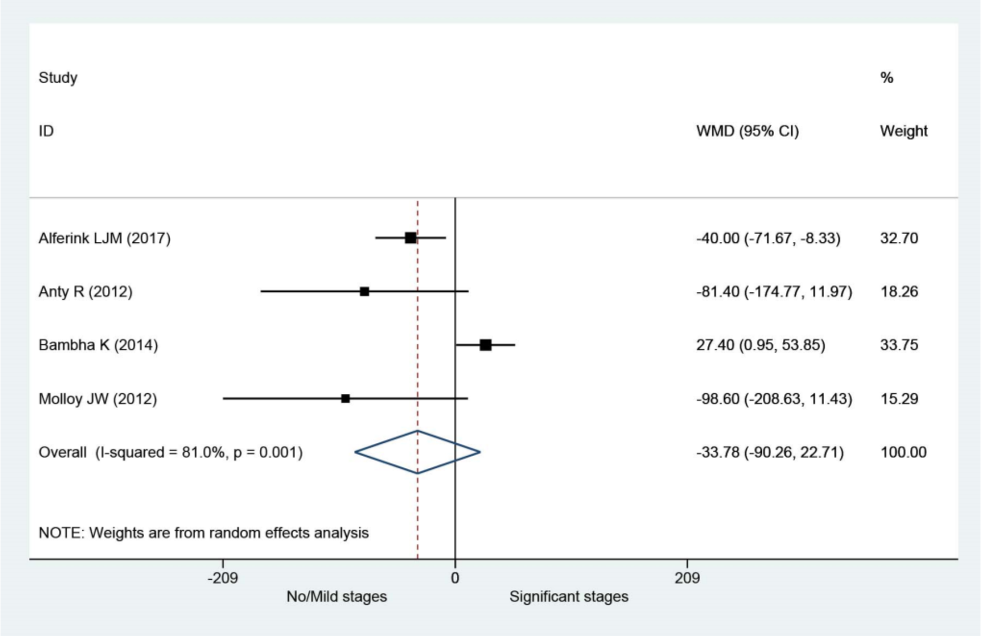

WMD, weighted mean difference

Supplementary Figure S2 Forest plot of coffee consumption (mg/day) of NAFLD patients with no/mild and significant stages of liver fibrosis
